# The prognostic value of the CRP-albumin ratio for peripheral artery disease: A cross-sectional study from the National Health and Nutrition Examination Survey

**DOI:** 10.1097/MD.0000000000048267

**Published:** 2026-04-10

**Authors:** Yujuan Wang, Lizhu Chen

**Affiliations:** a Department of Cardiovascular Surgery, Haikou Affiliated Hospital of Central South University Xiangya School of Medicine, Haikou, Hainan, China.

**Keywords:** ankle-brachial index, cross-sectional study, CRP-albumin ratio, National Health and Nutrition Examination Survey, peripheral arterial disease

## Abstract

The CRP-to-albumin ratio (CAR) has emerged as a composite inflammatory-nutritional biomarker with prognostic value in various cardiovascular conditions, yet its association with peripheral artery disease (PAD) in the general population remains underexplored. This study aimed to investigate the association between CAR and PAD in a nationally representative sample of US adults. We conducted a cross-sectional analysis of 6128 participants aged ≥40 years from the National Health and Nutrition Examination Survey (NHANES, 1999–2004). PAD was defined as an ankle-brachial index ≤0.90. Multivariable logistic regression models were used to evaluate the independent association between CAR and PAD, with progressive adjustment for demographic, behavioral, metabolic, and clinical covariates. A significant dose-response relationship was observed between CAR quartiles and PAD prevalence (*P*-trend < .001). After full adjustment, participants in the highest CAR quartile had more than twice the odds of PAD compared to the lowest quartile (odds ratios [OR]: 2.11, 95% confidence interval [CI]: 1.46–3.06). Subgroup analysis revealed a stronger association in individuals aged <65 years (*P*-interaction = .029). CAR is independently associated with PAD and may serve as an accessible biomarker for improving risk stratification and early detection, particularly in younger adults.

## 1. Introduction

Peripheral artery disease (PAD), a prevalent manifestation of systemic atherosclerosis, affects over 200 million people globally and is associated with significant morbidity, mortality, and impaired quality of life.^[1,2]^ The ankle-brachial index (ABI) remains a cornerstone for noninvasive diagnosis, with values ≤0.90 indicating PAD.^[3]^ Despite its clinical utility, ABI is underutilized in primary care settings, highlighting an unmet need for readily accessible biomarkers that could aid in risk stratification and early identification of high-risk individuals.^[4,5]^

The pathogenesis of PAD is intrinsically linked to chronic inflammation and oxidative stress, which drive atherosclerotic plaque progression and instability.^[6,7]^ C-reactive protein (CRP), a classical acute-phase reactant, is a well-established biomarker of systemic inflammation and has been consistently associated with the presence and severity of PAD in numerous epidemiological studies.^[8,9]^ Conversely, serum albumin, synthesized by the liver, is not only a indicator of nutritional status but also possesses anti-inflammatory, antioxidant, and endothelial-protective properties.^[10,11]^ Hypoalbuminemia has been independently linked to an increased risk of cardiovascular events and prevalent PAD, likely reflecting both malnutrition and chronic inflammatory states.^[12,13]^

The CRP-to-albumin ratio (CAR) integrates these 2 opposing pathways into a single, comprehensive metric, theoretically providing a more robust reflection of the balance between pro-inflammatory and anti-inflammatory/homeostatic processes.^[14]^ This novel ratio has demonstrated superior prognostic value compared to its individual components in various clinical contexts, including sepsis, various cancers, and coronary artery disease.^[15-17]^ For instance, a high CAR powerfully predicts mortality in patients with critical illness and poor long-term outcomes in those undergoing percutaneous coronary interventions.^[18,19]^

However, the specific utility of CAR in the context of PAD remains underexplored. While several studies have examined CRP or albumin independently in relation to PAD,^[20]^ evidence synthesizing their combined effect through the CAR is scarce and limited to specific, often severe, patient cohorts, such as those with critical limb ischemia or undergoing vascular surgery.^[18,19]^ A significant gap exists in understanding the association between CAR and PAD prevalence in a broad, representative general population. Such an investigation could establish CAR as a widely applicable tool for primary and secondary prevention strategies.

To address this knowledge gap, we conducted a cross-sectional analysis utilizing data from the National Health and Nutrition Examination Survey (NHANES), a nationally representative sample of the non-institutionalized US population. We hypothesized that a higher CAR would be independently associated with an increased prevalence of PAD. By leveraging the rigorous sampling methodology and extensive biomarker data of NHANES, this study aims to provide robust, generalizable evidence on the prognostic value of this easily derivable inflammatory-nutritional biomarker for PAD screening and risk assessment.

## 2. Materials and methods

### 2.1. Study population

This retrospective cross-sectional analysis utilized data from the 1999 to 2004 cycles of NHANES, a nationally representative program conducted by the National Center for Health Statistics (NCHS). The survey employs a stratified, multistage probability sampling design to produce estimates generalizable to the non-institutionalized US population.^[21]^ The NHANES protocol was approved by the NCHS Ethics Review Board, and written informed consent was obtained from all participants.^[22]^ For this secondary analysis of de-identified public data, no additional institutional review board approval was required.

From an initial pool of 31,126 participants, we excluded those under 40 years of age (n = 21,156) as ABI measurements were not performed in this group. We further excluded individuals with missing ABI data (n = 2399), ABI values > 1.4 (n = 73, indicating non-compressible vessels), and missing CRP or albumin measurements (n = 361). After additional exclusions for incomplete covariate data (n = 1049), the final analytic sample included 6128 adults with complete information for all study variables.

### 2.2. Peripheral artery disease

PAD was assessed using theABI according to standardized protocols outlined in contemporary vascular guidelines. Measurements were conducted exclusively in participants aged 40 years and older. Following a standardized 5-minute supine rest period, systolic blood pressures were obtained oscillometrically: first in the brachial arteries (with the higher value used as the reference), followed by bilateral measurements of the posterior tibial and dorsalis pedis arteries. To enhance precision, duplicate measurements were performed and averaged for all arterial sites in participants aged 40 to 59 years, while a single measurement was taken per site in those aged 60 years or older.^[21]^

The ABI was calculated for each lower extremity by dividing the highest ankle systolic pressure (either posterior tibial or dorsalis pedis) by the higher of the 2 brachial systolic pressures. The final ABI value for each participant was determined based on the lower of the 2 limb-specific ratios. Consistent with current international diagnostic standards, PAD was defined as an ABI value of ≤0.90 in either limb.^[23,24]^ This methodological approach ensured accurate and reproducible identification of hemodynamically significant arterial stenosis while accounting for age-related procedural variations within a large population-based sample.

### 2.3. Study variables and outcome

CAR defined as serum CRP divided by albumin, is a composite biomarker reflecting systemic inflammatory activity and nutritional status.^[25]^

Sociodemographic and lifestyle information was collected using standardized questionnaires. Clinical and biochemical parameters, including body mass index (BMI), blood pressure, and laboratory values, were obtained through examinations conducted in the Mobile Examination Center. Covariates were selected based on established literatures^[26,27]^ and included age, sex, race/ethnicity, educational attainment, marital status, family income, smoking status, physical activity level, BMI, total cholesterol, glycated hemoglobin, and comorbidities such as diabetes, hypertension, and cardiovascular disease. Detailed measurement methods and variable definitions are available on the NHANES website (www.cdc.gov/nchs/nhanes/; accessed on May 1, 2022).

Family income was categorized using the poverty–income ratio (PIR) into low (PIR ≤ 1.3), medium (PIR > 1.3–3.5), and high (PIR > 3.5) groups. Smoking status was classified as never, current, or former smoker, consistent with previous studies. Physical activity was assessed based on self-reported engagement in moderate (e.g., golfing or leisurely biking) or vigorous (e.g., swimming or high-speed cycling) activities lasting at least 10 minutes during the previous month. Participants were grouped into below-moderate (no moderate or vigorous activity), moderate (moderate but no vigorous activity), or high-intensity (any vigorous activity) categories.

Hypertension was defined as having an average systolic blood pressure or average diastolic blood pressure of at least 140/90 mm Hg, currently using antihypertensive drugs, having received a diagnosis from a healthcare professional, or any combination of these factors. Diabetes was defined by a fasting glucose level >7 mmol/L, a random glucose level of 11.1 mmol/L or higher, a glycated hemoglobin A1c of 6.5% or more, the use of medications to lower blood sugar, or a documented history of diabetes. The occurrence of cardiovascular diseases was assessed based on individuals’ self-reported experiences with congestive heart failure, coronary heart disease, angina pectoris, myocardial infarction, and stroke. BMI was calculated as weight in kilograms divided by height in meters squared.

### 2.4. Statistical analysis

Categorical variables are summarized as frequencies and percentages, while continuous variables are expressed as means with standard deviations or medians with interquartile ranges, depending on their distribution. Group comparisons were conducted using appropriate statistical tests: the Student *t* test or Mann–Whitney *U* test for continuous variables, and the chi-square test for categorical variables.

The relationship between CAR and PAD was evaluated using a series of multivariable logistic regression models with sequential adjustment for potential confounders. Model 1 was unadjusted; model 2 included demographic and socioeconomic factors (age, gender, race, education level, marital status, PIR), health behaviors (physical activity); model 3 further incorporated metabolic parameters (BMI, total cholesterol, glycated hemoglobin), health behaviors (smoking status); model 4 added clinical comorbidities (hypertension, diabetes, cardiovascular disease). Results are presented as adjusted odds ratios (OR) with corresponding 95% confidence interval (CI).

Subgroup analyses were performed to evaluate the consistency of associations across populations stratified by demographic and clinical characteristics. All analyses were conducted using R Statistical Software (V4.2.2, http://www.R-project.org, The R Foundation) and Free Statistics Analysis Platform (V1.9, Beijing, China, http://www.clinicalscientists.cn/freestatistics), with comprehensive methodological documentation ensuring transparency and reproducibility throughout the analytical workflow.

## 3. Results

### 3.1. Characteristics of the study population

**Figure 1.**
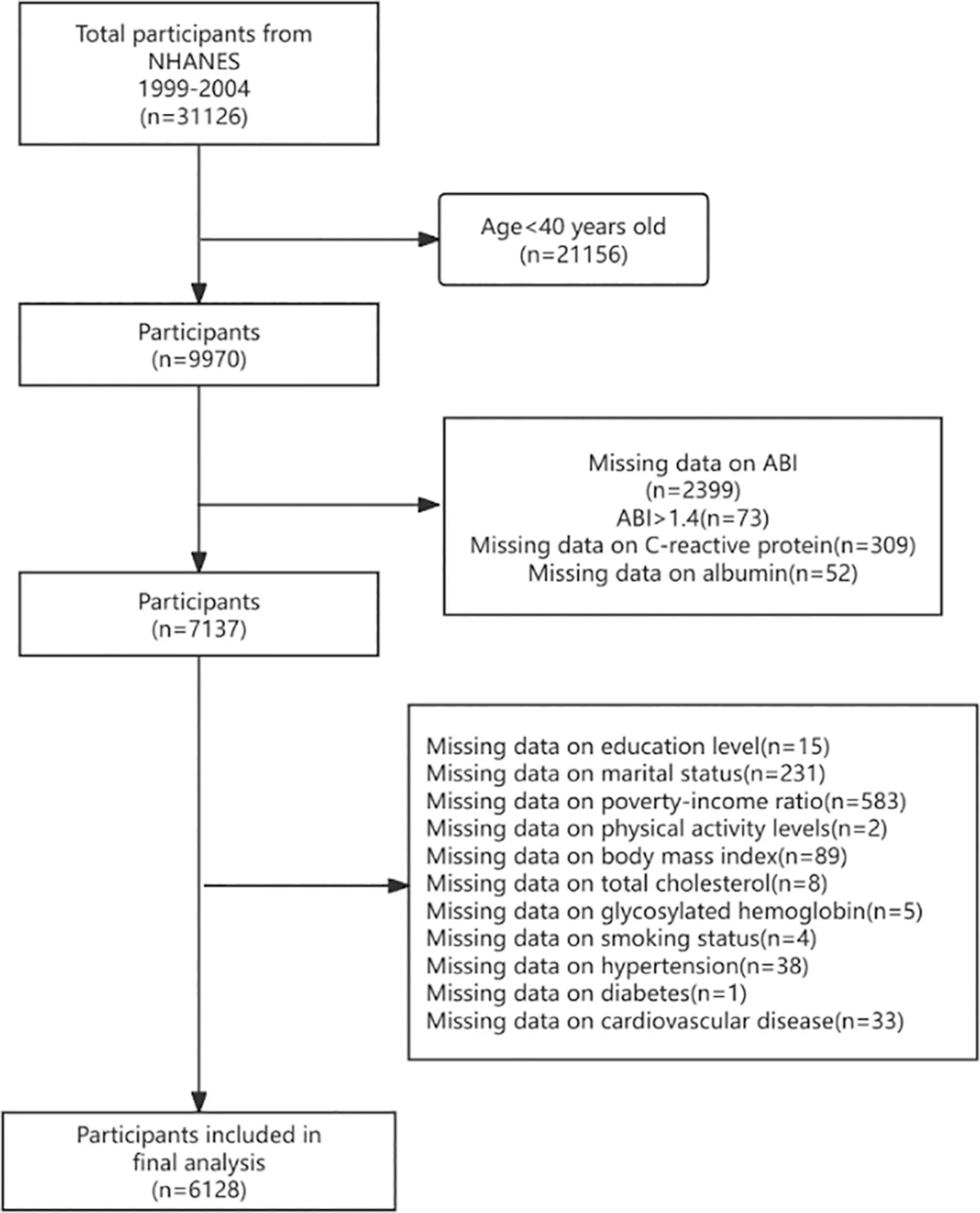
Flowchart of the study population selection from NHANES 1999–2004. ABI = ankle-brachial index, NHANES = National Health and Nutrition Examination Survey.

After applying sequential exclusion criteria to the initial 31,126 interviewed participants, the final analytical cohort consisted of 6128 individuals (Fig. 1). As summarized in Table 1, all baseline characteristics exhibited significant variation across CAR quartiles (all *P* < .001 unless indicated). A pronounced dose-response relationship was observed between CAR and PAD, with prevalence increasing from 3.1% in the lowest quartile (Q1) to 8.6% in the highest (Q4).

**Table 1:** Baseline characteristics stratified by CAR quartiles (Q).

Variables	Total					*P*-value
	(n = 6128)	Q1 (n = 1531)	Q2 (n = 1525)	Q3 (n = 1540)	Q4 (n = 1532)	
Gender, n (%)						<.001
Male	3141 (51.3)	931 (60.8)	892 (58.5)	745 (48.4)	573 (37.4)	
Female	2987 (48.7)	600 (39.2)	633 (41.5)	795 (51.6)	959 (62.6)	
Age (yr), mean ± SD	59.9 ± 13.0	57.8 ± 13.2	60.4 ± 13.3	61.1 ± 13.0	60.2 ± 12.3	<.001
Race, n (%)						<.001
Mexican American	1271 (20.7)	277 (18.1)	336 (22)	325 (21.1)	333 (21.7)	
Other Hispanic	236 (3.9)	49 (3.2)	61 (4)	66 (4.3)	60 (3.9)	
Non-Hispanic White	3405 (55.6)	914 (59.7)	884 (58)	860 (55.8)	747 (48.8)	
Non-Hispanic Black	1042 (17.0)	224 (14.6)	211 (13.8)	255 (16.6)	352 (23)	
Other races	174 (2.8)	67 (4.4)	33 (2.2)	34 (2.2)	40 (2.6)	
Education level, n (%)						<.001
Less than high school	1053 (17.2)	198 (12.9)	300 (19.7)	277 (18)	278 (18.1)	
High school diploma or GED	2366 (38.6)	559 (36.5)	548 (35.9)	575 (37.3)	684 (44.6)	
More than high school	2709 (44.2)	774 (50.6)	677 (44.4)	688 (44.7)	570 (37.2)	
MS, n (%)						<.001
Married/Living with partner	4072 (66.4)	1099 (71.8)	1054 (69.1)	1011 (65.6)	908 (59.3)	
Widowed/divorced/separated	1711 (27.9)	345 (22.5)	395 (25.9)	444 (28.8)	527 (34.4)	
Never married	345 (5.6)	87 (5.7)	76 (5)	85 (5.5)	97 (6.3)	
PIR, n (%)						<.001
Low income	1537 (25.1)	303 (19.8)	364 (23.9)	397 (25.8)	473 (30.9)	
Medium income	2321 (37.9)	542 (35.4)	589 (38.6)	596 (38.7)	594 (38.8)	
High income	2270 (37.0)	686 (44.8)	572 (37.5)	547 (35.5)	465 (30.4)	
Physical activity, n (%)						<.001
Sedentary	2767 (45.2)	578 (37.8)	670 (43.9)	683 (44.4)	836 (54.6)	
Moderate	1937 (31.6)	469 (30.6)	495 (32.5)	522 (33.9)	451 (29.4)	
Vigorous	1424 (23.2)	484 (31.6)	360 (23.6)	335 (21.8)	245 (16)	
BMI, kg/m^2^, mean ± SD	28.5 ± 5.6	25.9 ± 4.3	27.7 ± 4.6	29.1 ± 5.2	31.1 ± 6.6	<.001
CRP, mg/dL, median (IQR)	0.2 (0.1, 0.5)	0.1 (0.0, 0.1)	0.2 (0.1, 0.2)	0.3 (0.3, 0.4)	0.9 (0.7, 1.4)	<.001
Total cholesterol, mg/dL, mean ± SD	209.2 ± 41.3	204.4 ± 40.0	209.3 ± 42.1	213.2 ± 40.7	209.8 ± 42.1	<.001
HbA1c, %, mean ± SD	5.8 ± 1.1	5.6 ± 0.9	5.7 ± 0.9	5.8 ± 1.2	6.0 ± 1.4	<.001
Albumin, g/dL, mean ± SD	4.3 ± 0.3	4.4 ± 0.3	4.3 ± 0.3	4.2 ± 0.3	4.1 ± 0.3	<.001
Smoking, n (%)						.004
Never	2842 (46.4)	744 (48.6)	694 (45.5)	694 (45.1)	710 (46.3)	
Former	2113 (34.5)	528 (34.5)	552 (36.2)	548 (35.6)	485 (31.7)	
Current	1173 (19.1)	259 (16.9)	279 (18.3)	298 (19.4)	337 (22)	
Hypertension, n (%)						<.001
** **No	3936 (64.2)	1127 (73.6)	1019 (66.8)	938 (60.9)	852 (55.6)	
Yes	2192 (35.8)	404 (26.4)	506 (33.2)	602 (39.1)	680 (44.4)	
Diabetes, n (%)						<.001
No	5342 (87.2)	1375 (89.8)	1366 (89.6)	1322 (85.8)	1279 (83.5)	
Yes	786 (12.8)	156 (10.2)	159 (10.4)	218 (14.2)	253 (16.5)	
CVD, n (%)						<.001
No	5193 (84.7)	1336 (87.3)	1288 (84.5)	1318 (85.6)	1251 (81.7)	
Yes	935 (15.3)	195 (12.7)	237 (15.5)	222 (14.4)	281 (18.3)	
PAD, n (%)						<.001
No	5763 (94.0)	1483 (96.9)	1450 (95.1)	1430 (92.9)	1400 (91.4)	
Yes	365 (6.0)	48 (3.1)	75 (4.9)	110 (7.1)	132 (8.6)	

Q, quartile,values are given as mean ± standard deviation,median (IQR),or numbers and percentages.

BMI = body mass index, CAR = CRP-to-albumin ratio, CRP = C-reactive protein, CVD = cardiovascular disease, GED = general educational development, HbA1c = glycosylated hemoglobin, MS = marital status, PAD = peripheral arterial disease, PIR = poverty–income ratio, SD = standard deviation.

Participants with elevated CAR were more likely to be female, older, and of non-Hispanic Black race. Higher CAR levels were also associated with lower educational attainment, reduced family income, and sedentary behavior. Clinically, ascending CAR quartiles showed progressively adverse metabolic and inflammatory profiles, including higher BMI, elevated CRP, increased glycated hemoglobin, and lower serum albumin. Comorbidities such as hypertension, diabetes, and cardiovascular disease were also significantly more prevalent in higher CAR groups.

These results demonstrate that CAR effectively integrates inflammatory, nutritional, and metabolic dimensions of health, capturing a broad spectrum of sociodemographic and clinical risk factors associated with PAD. The strong graded relationship supports CAR’s potential utility as a composite biomarker for identifying high-risk individuals.

### 3.2. Association between CAR and PAD

Table 2 presents the multivariate associations between various covariates and PAD risk. The CAR demonstrated a strong independent association with PAD (OR: 2.19, 95% CI: 1.56–3.07, *P* < .001). Other significant risk factors included advanced age, non-Hispanic Black race, hypertension, diabetes, cardiovascular disease, and smoking (all *P* < .001). Protective factors included higher education, increased physical activity, and elevated albumin levels. These results underscore the value of CAR as an integrative biomarker and highlight multifactorial contributors to PAD pathogenesis.

**Table 2:** Association of covariates and PAD risk.

Variable	OR (95% CI)	*P*-value
Gender, n (%)		
Male	1 (reference)	
Female	1.02 (0.83–1.27)	.822
Age (yr), mean ± SD	1.08 (1.07–1.09)	<.001
Race, n (%)		
Mexican American	1 (reference)	
Other Hispanic	0.8 (0.39–1.64)	.541
Non-Hispanic white	1.3 (0.97–1.75)	.082
Non-Hispanic black	1.79 (1.27–2.52)	.001
Other races	0.6 (0.24–1.51)	.275
Education level, n (%)		
Less than high school	1 (reference)	
High school diploma or GED	0.7 (0.53–0.91)	.007
More than high school	0.43 (0.32–0.56)	<.001
MS, n (%)		
Married/living with partner	1 (reference)	
Widowed/divorced/separated	1.95 (1.57–2.43)	<.001
Never married	0.58 (0.3–1.11)	.099
PIR, n (%)		
Low income	1 (reference)	
Medium income	0.81 (0.63–1.03)	.081
High income	0.41 (0.31–0.55)	<.001
Physical activity, n (%)		
Sedentary	1 (reference)	
Moderate	0.58 (0.46–0.74)	<.001
Vigorous	0.28 (0.19–0.4)	<.001
BMI, kg/m^2^, mean ± SD	0.99 (0.97–1.01)	.163
Total cholesterol, mg/dL, mean ± SD	1 (1–1)	.126
CRP, mg/dL, median (IQR)	1.23 (1.13–1.34)	<.001
HbA1c, %, mean ± SD	1.21 (1.13–1.29)	<.001
Albumin, g/dL, mean ± SD	0.31 (0.23–0.43)	<.001
Smoking, n (%)		
Never	1 (reference)	
Former	1.93 (1.51–2.46)	<.001
Current	1.93 (1.45–2.57)	<.001
Hypertension, n (%)		
No	1 (reference)	
Yes	2.81 (2.27–3.49)	<.001
Diabetes, n (%)		
No	1 (reference)	
Yes	2.46 (1.92–3.16)	<.001
CVD, n (%)		
No	1 (reference)	
Yes	3.1 (2.46–3.9)	<.001
CRP-albumin ratio	2.19 (1.56–3.07)	<.001

BMI = body mass index, CRP = C-reactive protein, CVD = cardiovascular disease, HbA1c = glycated haemoglobin, PAD = peripheral artery disease, SD = standard deviation.

As shown in Table 3, a significant dose-response relationship was observed between CAR and the prevalence of PAD across all models. In the fully adjusted model (model 4), each quartile increase in CAR was associated with a 29% elevated risk of PAD (OR: 1.29; 95% CI: 1.15–1.45; *P* < .001). Participants in the highest CAR quartile (Q4) had more than twice the risk of PAD compared to those in the lowest quartile (OR: 2.11; 95% CI: 1.46–3.06; *P* < .001). This association remained robust after sequential adjustment for demographic, socioeconomic, behavioral, metabolic, and clinical confounders. The persistent gradient and statistical significance across progressive adjustments support CAR as a strong and independent biomarker associated with PAD.

**Table 3:** Association between CAR and PAD.

Variable	CRP-albumin ratio	*P*-value	Q1 (0.002–0.025)	Q2 (0.026–0.056)	*P*-value	Q3 (0.057–0.126)	*P*-value	Q4 (0.127–8)	*P*-value	Trend. test	*P*-value
	OR (95% CI)		OR (95% CI)	OR (95% CI)		OR (95% CI)		OR (95% CI)			
Model 1	2.19 (1.56–3.07)	<.001	1 (Ref)	1.60 (1.10–2.31)	.013	2.38 (1.68–3.36)	<.001	2.91 (2.08–4.09)	<.001	1.41 (1.28–1.56)	<.001
Model 2	1.91 (1.33–2.75)	<.001	1(Ref)	1.30 (0.89–1.91)	.176	1.89 (1.31–2.7)	.001	2.40 (1.68–3.43)	<.001	1.35 (1.21–1.5)	<.001
Model 3	1.67 (1.15–2.42)	.007	1(Ref)	1.25 (0.85–1.83)	.264	1.77 (1.22–2.56)	.003	2.15 (1.49–3.11)	<.001	1.30 (1.16–1.46)	<.001
Model 4	1.60 (1.11–2.31)	.012	1(Ref)	1.24 (0.84–1.83)	.277	1.74 (1.2–2.52)	.004	2.11 (1.46–3.06)	<.001	1.29 (1.15–1.45)	<.001

Model 1: adjusted for none.

Model 2: adjusted for gender + race + education level + MS + PIR + physical activity.

Model 3: adjusted for model 2 + BMI + total cholesterol + HbA1c + smoking status.

Model 4: adjusted for model 3 + cardiovascular disease + hypertension + diabetes.

BMI = body mass index, CI = confidence interval, CRP = C-reactive protein, HbA1c = glycated haemoglobin, MS = marital status, OR = odds ratio, PAD = peripheral artery disease, PIR = poverty–income ratio, Q = quartile, Ref = reference.

**Figure 2.**
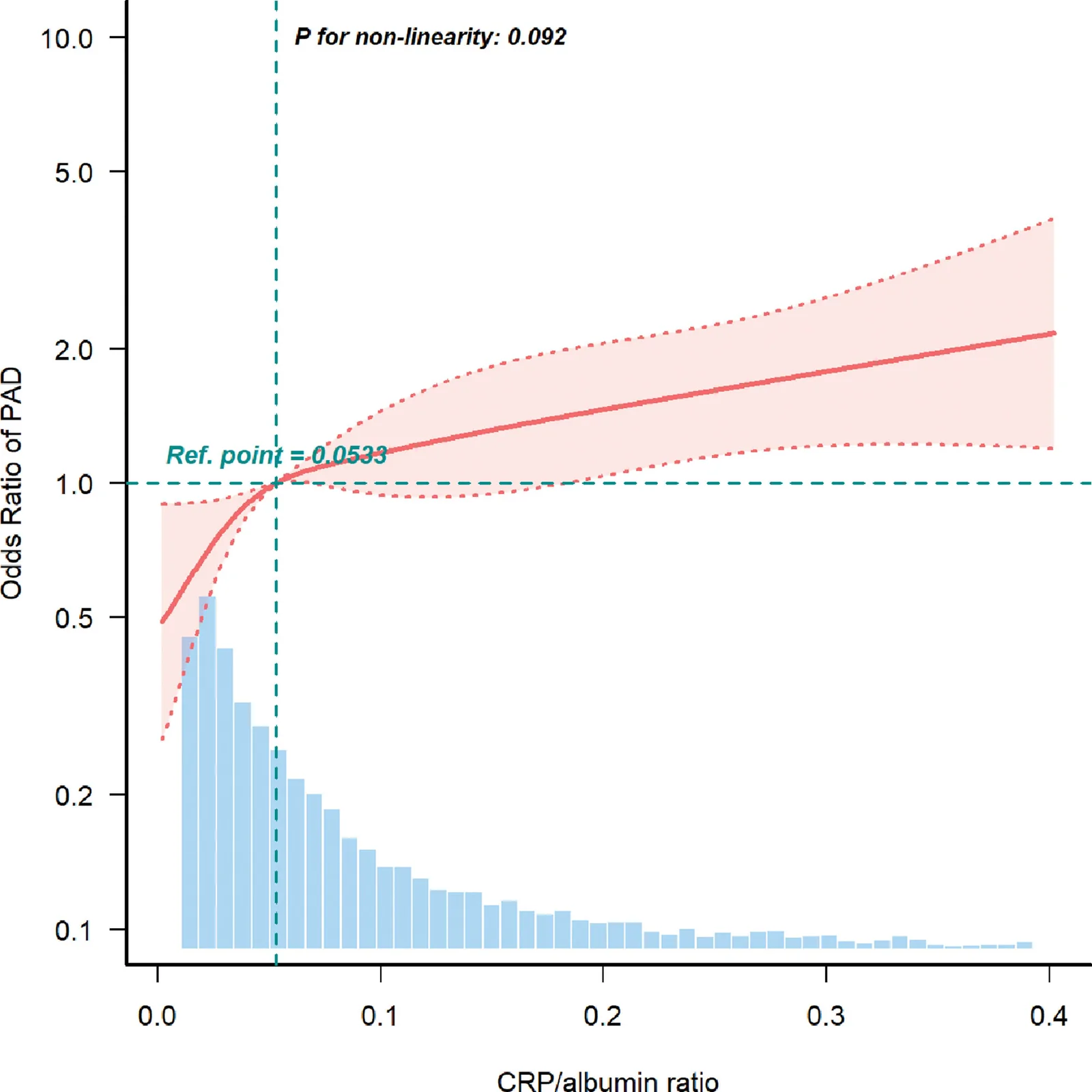
Restricted cubic spline analysis of the association between CAR and PAD. The solid line represents adjusted odds ratios, and the shaded area represents the 95% confidence interval. The model was adjusted for age, sex, race, education, marital status, poverty–income ratio, physical activity, smoking status, body mass index, total cholesterol, glycated hemoglobin, hypertension, diabetes, and cardiovascular disease. *P* for nonlinearity = .092. BMI = body mass index, CAR = CRP-to-albumin ratio, CI = confidence interval, OR = odds ratio, PAD = peripheral artery disease, PIR = poverty–income ratio.

Figure 2 illustrates the dose–response relationship between CAR and the odds of PAD using restricted cubic splines. A nearlinear positive association was observed, with increasing CAR values corresponding to elevated PAD risk (*P* for nonlinearity = 0.092). The results suggest a monotonic increase in PAD odds across the range of CAR, supporting its role as a continuous risk indicator.

### 3.3. Stratification analysis

**Figure 3.**
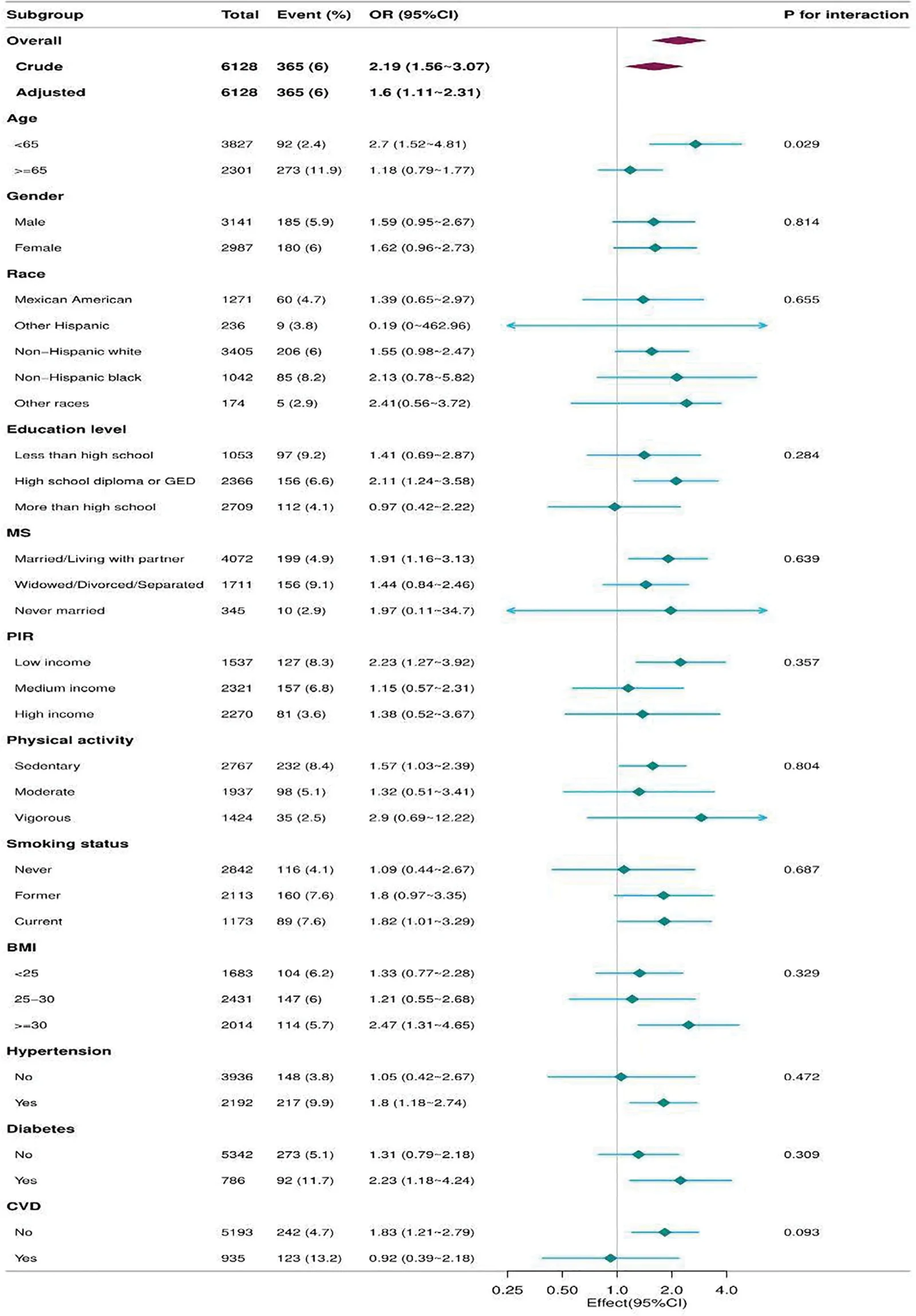
Subgroup analysis of the association between CAR (per 1-unit increase) and PAD. Odds ratios and 95% confidence intervals are shown for each subgroup, adjusted for all covariates except the stratification variable. The *P*-value for interaction is displayed for each subgroup comparison. CAR = CRP-to-albumin ratio, PAD = peripheral artery disease.

Figure 3 presents the association between CAR and PAD across various subgroups. A significant interaction was observed only for age (*P*-interaction = .029), with a stronger association among participants aged <65 years (OR: 2.70; 95% CI: 1.52–4.81) compared to those ≥65 years (OR: 1.18; 95% CI: 0.78–1.77). The association remained consistent across other demographic, socioeconomic, and clinical subgroups, including gender, race, education, income, physical activity, smoking status, BMI, and comorbidity status (all *P*-interaction > .05). These findings suggest that the CAR is a robust biomarker for PAD risk, particularly in younger adults.

## 4. Discussion

This study identified a significant, dose-dependent association between CAR and the prevalence of PAD among US adults, independent of conventional cardiovascular risk factors. The graded increase in PAD risk across CAR quartiles underscores CAR’s potential as a composite inflammatory-nutritional biomarker for early identification of high-risk individuals.

Our findings align with numerous studies that have established CAR as a prognostic marker across cardiovascular conditions. Consistent with our results, Kaplangoray et al demonstrated that CAR was a strong independent predictor of high thrombus burden in STEMI patients (OR: 10.206; 95% CI: 2.987–34.872, *P* < .001).^[28]^ Ardahanli and Özmen reported that CAR was significantly elevated in patients with coronary slow flow phenomenon, further supporting its role as a sensitive inflammatory marker in vascular pathology.^[29]^ Similarly, Yildirim et al reported that CAR was significantly higher in patients with severe carotid stenosis (0.56 ± 0.25 vs 0.14 ± 0.01, *P* < .001) and was an independent predictor of severe disease (OR: 1.051; 95% CI: 1.027–1.076).^[30]^ Cerşit et al found CAR to be an independent predictor of abdominal aortic aneurysm presence (OR: 3.162; 95% CI: 1.690–5.126, *P* = .001).^[31]^ The strong correlation between CAR and PAD observed in our analysis is further supported by Süleymanoğlu et al, who identified CAR as an independent predictor of amputation and mortality in PAD patients following endovascular intervention (OR: 2.23 for amputation, 95% CI: 1.27–3.92).^[19]^ Notably, however, our results contrast with those of Wu et al, who found that an alternative index was inversely associated with PAD (OR: 0.813, 95% CI: 0.717–0.923).^[32]^ This discrepancy may stem from differences in biomarker construct, as the inclusion of lymphocytes may reflect immune activation rather than pure inflammatory-nutritional imbalance. Additional studies by Özcan et al further validate CAR’s prognostic value in pulmonary embolism,^[33]^ while Söğüt et al demonstrated its utility in acute coronary syndromes.^[34]^ Luo et al highlighted its role as a nutrition-immunity-inflammation marker in elderly mortality.^[35]^ Tasbulak et al identified CAR as a novel marker indicating restenosis in superficial femoral artery,^[36]^ and Bayram et al demonstrated its prognostic value in carotid endarterectomy patients.^[37]^ The consistency of CAR’s predictive value across subgroups in our study – particularly its strong association in younger adults – further highlights its broad applicability.

The restricted cubic spline model revealed a near-linear relationship between CAR and PAD risk (*P* for nonlinearity = 0.092), suggesting a continuous increase in risk without an apparent threshold. Biologically, CAR encapsulates 2 fundamental pathways in atherogenesis: systemic inflammation and impaired endothelial protection and antioxidant capacity.^[38]^ CRP, a classical acute-phase reactant, directly promotes endothelial dysfunction by reducing nitric oxide bioavailability and increasing expression of adhesion molecules, thereby facilitating monocyte adhesion and transmigration into the intima.^[39]^ Within the arterial wall, CRP stimulates macrophage uptake of oxidized LDL, promoting foam cell formation and plaque instability.^[40]^ Conversely, albumin exerts multiple atheroprotective effects: it binds and transports bioactive lipids, inhibits platelet aggregation, scavenges reactive oxygen species, and preserves endothelial glycocalyx integrity.^[41]^ Hypoalbuminemia thus reflects not only malnutrition but also a state of heightened oxidative stress and impaired vascular repair capacity.^[11,42,43]^ The combination of elevated CRP and low albumin – captured by CAR – may therefore represent a synergistic pro-atherogenic state in which inflammation is amplified while protective mechanisms are simultaneously compromised. This is particularly detrimental in peripheral arteries, which are exposed to disturbed blood flow and oscillatory shear stress, rendering them susceptible to early atherosclerotic changes and progression.^[44]^ The prognostic value of CAR extends beyond PAD, as it has also been shown to predict the severity of coronary artery disease and ischemia while Sabanoglu et al demonstrated its ability to predict severity of coronary artery disease and ischemia^[45,46]^ and to be associated with radial artery thrombosis following transradial angiography.^[47]^

Key strengths of this study include the large, nationally representative NHANES cohort, standardized ABI measurements, extensive adjustment for confounders, and consistency across multiple sensitivity analyses.^[48]^ Several limitations should be considered when interpreting our findings. First, the cross-sectional design of this study precludes any causal inference regarding the relationship between CAR and PAD. Although we observed a consistent and independent association, reverse causality cannot be ruled out; it is possible that subclinical PAD or its risk factors may influence inflammatory and nutritional status. Second, CRP and albumin were measured at a single time point, which may not accurately reflect long-term inflammatory or nutritional exposure. Chronic inflammation and hypoalbuminemia are dynamic processes, and single measurements could lead to misclassification of exposure and may underestimate or overestimate the true association. Future longitudinal studies with serial biomarker assessments are warranted to confirm the temporal relationship and to evaluate whether changes in CAR over time predict PAD incidence.

While our findings support CAR as a robust and independent biomarker for PAD, its translation into clinical practice requires careful consideration. The continuous nature of the association, as illustrated by the restricted cubic spline analysis (Fig. 2), suggests that any dichotomous threshold would be arbitrary without prospective validation. Future studies should aim to establish clinically meaningful cutoff values in diverse populations and assess whether adding CAR to existing risk stratification tools (e.g., Framingham Risk Score, ABI-based algorithms) improves risk discrimination and reclassification. Moreover, although CAR is inexpensive and readily calculated from routine laboratory tests, its incremental value beyond established risk factors must be rigorously evaluated in outcome-based studies before routine clinical use can be recommended. If validated, CAR could serve as an adjunctive screening tool to identify high-risk individuals who may benefit from targeted lifestyle interventions or further vascular assessment.

## 5. Conclusions

CAR is a strong and independent biomarker associated with PAD, reflecting intertwined inflammatory and nutritional deficits. Its widespread availability and low cost support its potential integration into routine cardiovascular risk assessment. Future prospective studies should validate its prognostic utility and evaluate whether CAR-guided management improves clinical outcomes in PAD patients.

## Acknowledgments

We express our gratitude to Qiang Liu from the Department of Cardiovascular Surgery at Peking University First Hospital Taiyuan Hospital for providing valuable assistance in statistical analysis, study design consultations, and offering insightful feedback on the manuscript.

## Author contributions

**Conceptualization:** Lizhu Chen.

**Data curation:** Yujuan Wang.

**Formal analysis:** Yujuan Wang.

**Investigation:** Lizhu Chen.

**Methodology:** Yujuan Wang.

**Supervision:** Lizhu Chen.

**Validation:** Lizhu Chen.

**Visualization:** Lizhu Chen.

**Writing – original draft:** Yujuan Wang, Lizhu Chen.

**Writing – review & editing:** Lizhu Chen.
